# The impact of ICDAS on occlusal caries treatment recommendations for high caries risk patients: an in vitro study

**DOI:** 10.1186/s12903-019-0730-8

**Published:** 2019-03-07

**Authors:** Muawia A. Qudeimat, Yacoub Altarakemah, Qasem Alomari, Nour Alshawaf, Eino Honkala

**Affiliations:** 1 Department of Developmental and Preventive Sciences, Faculty of Dentistry, Kuwait University, P.O.Box: 24923, Safat-13110 Kuwait, Kuwait; 2 Department of Restorative Sciences, Kuwait University, Kuwait, Kuwait; 3 Department of General Dental Practice, Kuwait University, Kuwait, Kuwait; 4 Institute of Clinical Dentistry, UiT, The Arctic University of Norway, Tromsø, Norway

**Keywords:** Dental caries, ICDAS, Clinical decision making, High risk, Caries lesion treatment

## Abstract

**Background:**

The diagnostic criteria and tools used in caries lesion detection have been shown to affect the decision-making for caries treatment. Compared to other diagnostic criteria/classifications, ICDAS has been shown to provide a more accurate method for the detection of occlusal caries lesions. The influence of using ICDAS on caries treatment recommendations has received increasing attention in recent years. Therefore, the aim of this study was to assess the impact of ICDAS on dentists’ occlusal caries lesions’ treatment decisions for patients at high risk for caries.

**Methods:**

Five dentists examined the occlusal surfaces of 270 extracted premolars and permanent molars. For a predetermined clinical scenario, the examiners were asked to indicate their treatment recommendations for each tooth. Four weeks later, all the examiners were trained and calibrated for the use of ICDAS. Then the investigators examined the same 270 teeth independently and indicated their treatment recommendations using the same clinical scenario. Histological validation was used to determine the caries lesions detection performance of the examiners using ICDAS and to assess the relationship between the presence of dentin caries and treatment recommendations for each examiner before and after ICDAS training. Specificity, sensitivity, area under the receiver operating characteristic curve (AUC), and Spearman’s correlation coefficients were calculated. The Wilcoxon two-related sample rank test was used to test for differences between treatment recommendations.

**Results:**

The strongest correlation for inter-examiner reproducibility was found between the ICDAS D2 cut-off point (ICDAS codes 3–6 as dentin caries) and histologic dentin caries. Treatment recommendations among different examiners before and after ICDAS training demonstrated a statistically significant increase in operative intervention and an increase in the percentage of overtreatment recommendations for two examiners.

**Conclusions:**

The impact of ICDAS on the examiners’ caries lesion treatment recommendations varied among the dentists in this study. Treatment decision-making can be influenced by the caries lesion’s detection and classification system used.

## Background

Accurate detection of occlusal caries lesions using conventional methods has inherent diagnostic uncertainties [1]. The introduction of the International Caries Detection and Assessment System (ICDAS) and the International Caries Classification and Management System (ICCMS™) provided an evidence-based method for comprehensive caries classification and management for dental practitioners and educators [2–4]. ICDAS has been shown to provide a more accurate detection and an improved sensitivity for the detection of occlusal caries lesions compared to other methods and tools [2, 3, 5, 6]. On the other hand, the main objectives for the ICCMS™ are to stage and assess the activity of the caries process which is followed by risk-adjusted preventive care, control of initial non-cavitated lesions, and conservative treatment for cavitated and deep dentinal caries lesions [4]. However, researchers have concluded that, although the use of ICDAS might improve and objectify caries lesion detection [7], similar to other methods, it is prone to over- and underdiagnoses [8–12].

Even with the increasing use of methods and tools that are more accurate for caries lesions’ detection among dental practitioners and researchers [13], the mechanisms or criteria dentists use for making caries treatment decisions are still not fully understood [14–16]. It has been found that variation in caries lesions’ treatment decisions may stem from several uncertainties, including ambiguity of caries diagnosis data and variations in interpretation [15, 17–19]. In addition, differences among dentists including knowledge, skill and assiduousness in conducting the examination, diagnostic criteria and tools employed, and beliefs about the course of the disease, risk factors for the disease, and treatment effectiveness have been suggested to contribute to variations in decision-making [15–20]. The harm from misdiagnosis of carious lesions strongly depends on the allocated treatment. While undertreatment represents a potential dental public health problem, overtreatment raises the costs of dental care and may have adverse effects on oral health [21]. The influence of ICDAS as a caries detection and classification system on the clinician’s caries treatment decision-making has received increasing attention in recent years [15, 16, 19]. Therefore, the aim of this study was to assess the impact of ICDAS on dentists’ caries lesion treatment decisions. The null hypothesis was that there is no difference in occlusal caries lesion treatment decisions among dentists before and after ICDAS training.

## Methods

Ethical approval was obtained from the Health Science Centre Ethical Committee, Kuwait University. Permanent teeth (270 premolars and molars) without interproximal caries lesions, fluorosis, marked tooth wear or staining, enamel/dentin developmental defects, fissure sealants, or restorations were selected from a pool of 651 extracted teeth. All of the teeth were stored in 0.1% thymol solution immediately after extraction. Soft tissue debris was removed, and the teeth were thoroughly cleaned. The teeth were then mounted in acrylic blocks. Each block was number coded to facilitate randomization of clinical examination and histological sectioning.

Five examiners participated in this study. Three examiners worked in the department of restorative sciences (examiner one: a scholarship dentist with more than five years of clinical experience; examiner two: a clinical associate professor with 19 years of clinical and academic experience; and examiner three: a clinical assistant professor with 10 years clinical and academic experience). The other two examiners worked in the department of developmental and preventive sciences (examiner four: a clinical associate professor with 20 years clinical and academic experience; and examiner five: a clinical professor with 38 years of clinical and academic experience). In addition, all the examiners were regularly involved in treating patients and training undergraduate students on caries lesions detection and treatment. This study was conducted between October 2012–September 2016 and at the time of carrying out the visual detection and treatment decision component of this study, none of the examiners had any information about the ICCMS™ .

In the first part of this study, the examiners were asked to independently evaluate the occlusal surfaces of the teeth and choose their treatment recommendation for each tooth from a list of options. No attempts were made to detect lesion activity. The list of treatment recommendations included the following: (1) no action, (2) non-operative care (fluoride toothpaste, regular recall visits, and/or professional topical fluoride and preventive or therapeutic fissure sealants), and (3) operative care (preventive resin restoration, amalgam/tooth-colored restoration, pulp therapy, and extraction). The examiners were instructed to consider the following statement for each tooth: “A healthy 12 year-old patient presents to your practice for his/her first dental appointment. After a thorough clinical evaluation, you arrive at an optimum comprehensive dental care plan for this patient. Knowing that the child is at high caries risk, the child and his parents are cooperative and that the cost of treatment should not be considered as a factor, how would you manage this tooth?”

Four weeks later, in the second part of the study, all the investigators read the criteria manual of the ICDAS scoring system and completed the ICDAS e-learning program [22]. The examiners were then calibrated by independently coding 30 extracted permanent teeth (excluded from the study sample). This was followed by a discussion (lead by the most senior examiner EH) to clarify any uncertainties. After one week of ICDAS training and calibration, all the investigators examined the study sample teeth independently, blinded to the scores of the other examiners. The investigators were instructed to record the highest/worst ICDAS score and chose their treatment recommendation (using the same patient scenario as above) for the occlusal surfaces of all of the teeth.

All the tooth examinations and treatment decisions were carried out under standard conditions using a dental light (A-dec 300, A-dec, Newberg, OR, USA) and 3:1 syringe to dry and wet the teeth as appropriate, with access to a blunt probe (CPITN). In between the examination sessions, the teeth were stored in 0.1% thymol solution except when actively dried for the examinations.

### Histological validation

At the end of the study, each acrylic block was sectioned in longitudinal bucco-lingual or mesio-distal planes through specific points of interest (the worst/highest ICDAS score as agreed upon by two investigators) with a water-cooled diamond disc on a cutting machine (IsoMet® Low Speed Saw, Buehler, Lake Bluff, IL, USA). All the teeth sections were separated from the acrylic block and numbered for examination. Two examiners, with prior experience in caries histological classification systems inspected each tooth section under 10 x magnification using a stereomicroscope (Leica MZ6, Leica Microsystems Wetzlar, GmbH, Wetzlar, Germany). Ekstrand et al.’s (1997) histological criteria was used to record caries lesion extension at each investigated site [23]. The highest histological score was reported for each tooth section. Both investigators examined the sections independently and were blinded to the other investigator’s score. In cases of disagreements, the sections were re-examined and a consensus reached.

### Statistical analysis

The merged-ICDAS scoring system (codes: 0, 1–2, 3–4, and 5–6) was used to evaluate inter-examiner reproducibility. Maximum kappa statistics (K_max_) were used to test for marginal homogeneity (bias for the inter-examiner scores) [24]. A heuristic maximum kappa of < 0.8 was indicative of examiner bias [10]. This was followed by assessment of the bivariate symmetry for inter-examiner’s reproducibility. Bivariate agreement between examiners was assessed using linear weighted kappa (K_L_) [25].

To determine the caries lesion detection performance of the examiners, the sensitivity, specificity, area under the Receiver Operating Characteristic (ROC) curve, and Spearman’s correlation coefficients for ICDAS mode scores of all the examiners were calculated. The ICDAS D1 threshold (code 0 as sound/enamel carious lesion and codes 1–6 as dentin carious lesion), ICDAS D2 threshold (codes 0–2 as sound/enamel carious lesion and codes 3–6 as dentin carious lesion), and ICDAS D3 threshold (codes 0–3 as sound/enamel carious lesion and codes 4–6 as dentin carious lesion) were compared to histological dentin caries (Ekstrand et al.’s histological code 2 and above).

The Wilcoxon two-related sample Rank Test was used to: (1) test for differences between the treatment recommendations made during the two sessions for each examiner, and (2) to assess the relationship between the presence of dentin caries lesion (histological code 2 and above) and the treatment recommendations for each examiner during the two sessions. SAS for Windows (version 9.3, SAS Institute Inc., Cary, NC, USA) was used for the data analysis. A probability level of less than 0.05 was considered statistically significant.

## Results

Of the 270 teeth included in this study, 207 were molars and 63 were premolars. Table 1 demonstrates the distribution of the merged-ICDAS scores for each examiner. The scores for inter-examiner reproducibility were not always comparable (Table 2). The linear weighted kappa statistics for the inter-examiner reproducibility ranged between 0.50–0.68. The lowest scores were found between examiner one and examiners three and five (linear weighted kappa of 0.5).

**Table 1:** Distribution of merged-ICDAS scores for 270 teeth among the five different examiners

Examiners	ICDAS Score (%)			
	0	1–2	3–4	5–6
1	8	43	37	12
2	22	50	15	13
3	24	51	13	12
4	23	25	37	15
5	34	31	23	12

**Table 2:** Inter-examiner reproducibility for merged-ICDAS visual examination

Examiners	1	2	3	4	5
1		0.59^a^	0.52	0.62	0.52
2	0.56^b^ 0.63^c^		0.70	0.56	0.61
3	0.50 0.60	0.68 0.95		0.54	0.61
4	0.61 0.79	0.56 0.75	0.54 0.71		0.55
5	0.50 0.63	0.60 0.81	0.60 0.81	0.56 0.72	

Above diagonal entry, ^a^observed agreement (P_O_); below diagonal entries, ^b^linear weighted kappa (K_L_), ^c^maximum kappa (K_max_)

The distribution of the study sample over the different histological scores is presented in Table 3. Sound teeth and those with caries lesions affecting the outer half of the enamel represented 34% of the sample. The weighted kappa value for the two examiners who assessed the histological section scores was 0.88. When studying the correlation between mode ICDAS score for all of the examiners with the histological findings at the dentin caries lesion level (code 2–4), the ICDAS D2 cut-off point (codes 0–2 as sound/enamel carious lesion and codes 3–6 as dentin carious lesion) demonstrated the strongest correlation (*p* < 0.05) (Table 4).

**Table 3:** Distribution of caries lesion extension using Ekstrand and others histological criteria

Histological code	Description	Number of teeth (%)
0	Sound surfaces	28 (10)
1	Demineralization in the outer half of the enamel	66 (24)
2	Demineralization involving the inner half of the enamel and outer third of the dentin	100 (37)
3	Demineralization involving the middle third of the dentin	30 (11)
4	Demineralization involving the inner third of the dentin	46 (17)
Total		270 (100)

**Table 4:** The correlation between mode ICDAS score and histological criteria at caries into dentin cut-off point (scores 2–4)

	ICDAS-D1^a^	ICDAS-D2^b^	ICDAS-D3^c^
Sensitivity	0.91	0.51	0.31
Specificity	0.49	0.97	0.99
Area under the ROC curve (Az)	0.70^*^	0.74^*^	0.65^*^
95% confidence interval	0.63–0.77	0.68–0.79	0.59–0.72
Standard error	0.04	0.03	0.03
Spearman’s correlation coefficient	0.45^*^	0.48^*^	0.35^*^

^a^ICDAS D1 = 0 sound/enamel carious lesion and codes 1–6 dentin carious lesion

^b^ICDAS D2 = codes 0–2 sound/enamel carious lesion and codes 3–6 dentin carious lesion

^c^ICDAS D3 = codes 0–3 sound/enamel carious lesion and codes 4–6 dentin carious lesion

^*^Correlation is significant (*p* < 0.05)

Treatment recommendations based on a dichotomized decision for at high caries risk patient among different examiners before ICDAS training and after ICDAS training are presented in Table 5. ICDAS training statistically significantly increased the percentages of operative recommendations for two examiners (*p* < 0.001).

**Table 5:** Percentages of management recommendations for high caries risk patients among the different examiners before and after ICDAS training

Before to ICDAS training			After ICDAS training	
Examiners	Non-Operative %	Operative %	Non-Operative %	Operative %
1^*^	55.5	44.5	32	68
2	73.5	26.5	72	28
3	78.5	21.5	74.5	25.5
4^*^	59	41	34.5	65.5
5	80	20	80.5	19.5

^*^Significant between before and after ICDAS training (*p* < 0.001)

The percentages of treatment recommendations in relation to histological dentin caries lesion (code 2 and above) for all the teeth in the high caries risk patients among the different examiners are presented in Tables 6 and 7. Statistically significant differences were present for two examiners between recommendations made before ICDAS training and recommendations made after ICDAS training. ICDAS training increased the percentage of overtreatment recommendations for two dentists.

**Table 6:** Percentages of management recommendations in relation to dentin caries lesion (histological codes 0–1 non-operative care)

Before ICDAS training				After ICDAS training		
Examiners	Appropriate	Under-treatment	Over-treatment	Appropriate	Under-treatment	Over-treatment
1^*^	74	23	3	77	10	13
2	58	40	2	61	38	1
3	56	44	0	59	40	1
4^*^	72	26	2	76	12	12
5	54	46	0	54	46	0

^*^Significant between before and after ICDAS training (*p* < 0.001)

**Table 7:** Percentages of management recommendations in relation to dentine caries lesion (histological codes 0–2 non-operative care)

Before ICDAS training				After ICDAS training		
Examiners	Appropriate	Under-treatment	Over-treatment	Appropriate	Under-treatment	Over-treatment
1^*^	79	2	19	57	2	41
2	84	9	7	88	6	6
3	87	10	3	85	9	6
4^*^	82	3	15	62	0	38
5	87	10	3	86	11	3

^*^Significant between before and after ICDAS training (*p* < 0.0001)

## Discussion

In recent years, the focus on caries lesion detection, diagnosis and management research has shifted from cavitated to non-cavitated lesions [5, 26]. ICDAS was mainly designed to identify clinical stages of the caries process, which precede cavitation. In addition, the introduction of this system aimed at helping epidemiologists, clinicians, and educators to make the best and most informed decisions about appropriate diagnosis, prognosis, and clinical management [4, 6].

It has been previously suggested that dentists’ caries lesion detection skills depend on the similarity of what is seen on clinical examination with presentations encountered previously that have been believed to be dental caries requiring treatment [14–16, 19]. A range of patient and practitioner factors have been reported and may affect decisions of a specific caries lesion detection and treatment practices. In this study, we investigated the impact of ICDAS on dentists’ caries lesion treatment decisions.

It has been postulated that many factors can influence the reproducibility results of the ICDAS scoring system including the prevalence of caries at different progression levels, the use of a small number of patients and investigators, the investigators’ education and training background, their clinical and research experiences, and other unexamined factors [10–12, 15, 16, 19, 27]. In this study, the weighted kappa for the inter-examiner reproducibility ranged between moderate to substantial agreement (0.50 and 0.68). This is lower than the previously reported acceptable value (> 0.80) for inter-examiner reproducibility [3, 28]. However, similar results for inter-examiner reproducibility using the ICDAS scoring system were reported in previous studies [8–12].

In the absence of substantiated clinical evidence, there is a tendency for clinicians to make treatment choices based on perceptual and judgmental variations rather than a rational weighing of the outcomes and probabilities [18, 29]. Perceptual variations occur when dentists’ treatment decisions differ due to their different perceptions of the condition they are facing [29]. The perceptual variation among the five examiners for caries lesion detection using ICDAS was evident in the current study where the marginal homogeneity analysis demonstrated statistically significant disagreements between the examiners for caries lesion detection. This is in agreement with findings from recent studies [9, 10, 12]. On the other hand, judgmental variations occur when dentists’ opinions about appropriate caries lesion treatment differ, even in cases in which their perception of the dental caries lesion diagnosis was similar [18, 20, 29]. The results of this study showed that before and after ICDAS training, three of the examiners selected more non-operative care as treatment recommendations compared to the other two examiners. It is possible that regardless of ICDAS, the three dentists perceived enamel carious lesions as sound or non-cavitated and therefore did not recommend operative care. This in agreement with a study that concluded that dentists leaned toward diagnosing teeth with enamel carious lesions as sound and teeth with non-cavitated dentinal caries as caries restricted to the enamel [9]. On the contrary, for two of the examiners, the operative and non-operative decisions for caries lesions’ treatment before and after ICDAS training were statistically significantly different. Compared to the other examiners, the same two examiners were able to identify higher percentages of ICDAS codes 3 and 4 (enamel breakdown with no dentin visible and a dentinal shadow with no cavitation into the dentin). This is in agreement with previous studies, that demonstrated that the choice of operative treatment was more when the teeth were scored as ICDAS codes 3 and 4 [19, 30].

In this study, when using the histological investigation to assess the appropriateness of the treatment decisions made by the examiners, it was found that when combining Ekstrand et al.’s (1997) [23] histological codes 0 and 1, caries lesions treatment recommendations were appropriately made in 54–74% of the teeth prior to ICDAS training compared to 54–77% after ICDAS training. Although the differences were statistically significant for some examiners, they were not clinically significant, as the percentage of teeth which received the appropriate treatment remained the same. When adding Ekstrand et al.’s [23] histological code 2 to codes 0 and 1, the dentists’ appropriate caries lesions treatment recommendations dropped from 79-87% to 57-88%. The differences were statistically and clinically significant for two of the examiners with a tendency for overtreatment. Banting et al. (2013) reported that when histologically examining teeth classified as ICDAS code 2, 44% of the teeth had demineralization involving between 50% of the enamel and 1/3 of the dentin and in 6% caries involved the inner third of the dentin [31]. In addition, in a study by Ekstrand et al. (1998), of 24 lesions graded as histological score 2, the majority (58%) had demineralization in the dentin [32]. It is possible that in this study, when making a treatment decision, two dentists viewed ICDAS code 2 as caries lesions involving dentin and decided to provide operative treatment, while the other three dentists considered it as caries lesion involving enamel and therefore did not affect their treatment choices. Diniz et al. (2011) reported that for ICDAS code 2, dentists made a decision to restore the tooth in one third of the sampled teeth [19]. It is also likely that when uncertainty is present regarding the extent of dental caries lesions, as in the case of ICDAS code 2, dentists use other tooth and/or patient information prior to a decision concerning the recommended intervention for a specific tooth. A patient’s caries risk assessment is one tool that is considered a cornerstone in patient-centered caries treatment decision-making [33, 34]. Since caries risk assessment for a patient determines the probability of new caries lesions and/or the change in the size or activity of the current lesions, this could have played a role in the decision for caries treatment recommendations in the current study [34]. Gomez et al. (2014) concluded that for ICDAS code 2, the odds of a high caries risk patient having operative treatment is higher than for a low caries risk patient [30]. Therefore, the impact of ICDAS training on the selection of the intervention decision (that is, operative vs non-operative) could have been different if the scenario was for a patient at low risk of developing caries.

Dentists cannot reliably detect all caries lesions using only visual/tactile techniques; a significant number of lesions will be missed [13, 35]. Other tools that have shown association with caries lesion detection and treatment recommendations include intraoral radiographs [13, 19], Quantitative Light-induced Fluorescence [36] and the ICDAS-Lesion Activity Assessment (LAA) scoring system [27, 37]. However, studies on the effect of adding radiographs and LAA to the visual/tactile examination on caries lesions detection and treatment recommendations provided inconsistent results [13, 19, 27, 37].

The limitations of this study include the fact that this investigation was performed under laboratory conditions and it is not possible to know if the examiners considered the clinical scenario during the assessment of the teeth. Moreover, all of the examiners performed the assessments before ICDAS training and then made the evaluations using ICDAS, and therefore it is not possible to know if the changes in treatment recommendations were strictly due to ICDAS or if there was an influence of the previous knowledge of the sample. Finally, it is possible that an in vivo study design using parallel groups of examiners, randomly allocated to two strategies, with a higher number of examiners and in conjunction with radiographs and the ICDAS-LAA would be necessary when patient’s oral health conditions are evident for the examiners.

## Conclusions

Treatment recommendations among different examiners after ICDAS training demonstrated a statistically significant increase in operative intervention and an increase in the percentage of overtreatment recommendations for two examiners.

## Abbreviations

ICCMS™: International Caries Classification and Management System™
ICDAS: International Caries Detection and Assessment System
